# Medical staff's perception of factors contributing to accelerated rehabilitation in patients with cervical spinal cord injury: A qualitative research

**DOI:** 10.1002/nop2.1715

**Published:** 2023-03-06

**Authors:** Qiuxue Li, Qianghuizi Zhang, Weiwei Liu, Zheyi Zhou

**Affiliations:** ^1^ School of Nursing Capital Medical University Beijing China; ^2^ AHPRA Melbourne Victoria Australia

**Keywords:** cervical spinal cord injury, faster recovery, nursing quality, physical therapy, qualitative research, rehabilitation

## Abstract

**Aim:**

To explore the factors affecting the quality of accelerated rehabilitation for patients with cervical spinal cord injury, therefore, to propose targeted improvement strategies and provide reference for promoting the quality of nursing care for accelerated rehabilitation.

**Design:**

This descriptive qualitative inquiry followed the COREQ guidelines.

**Methods:**

From December 2020 to April 2021, 16 subjects including orthopaedic nurses, nursing management experts, orthopaedic surgeons, anaesthesiologists and physical therapists experienced in accelerated rehabilitation were selected by objective sampling method to conduct semi‐structured interviews. Thematic analysis was used to analyse the interview content.

**Results:**

Through analysis and summary of the interview data, the following two themes and nine sub‐themes were finally extracted. Factors related to the quality of accelerated rehabilitation structure include: construction of multidisciplinary teams, thorough system guarantee and adequate staffing. Factors related to the quality of accelerated rehabilitation process include: inadequate training and assessment, lack of medical staff's awareness, incapability of the accelerated rehabilitation team members, poor multidisciplinary communication and collaboration, lack of patient's awareness, and ineffective health education.

**Conclusion:**

Quality of implementation of accelerated rehabilitation can be improved by maximizing the role of multidisciplinary team, establishing a flawless accelerated rehabilitation system, increasing allocation of nursing resources, enhancing knowledge of medical staff, improving their awareness of accelerated rehabilitation, establishing personalized clinical pathways of accelerated rehabilitation, increasing communication and collaboration among multidisciplinary disciplines, and improving health education of patients.

## 1 INTRODUCTION

Enhanced Recovery After Surgery (ERAS) was first proposed by Danish surgeon Henrik Kehlet at the American Annual Meeting of Surgery in 1997. It refers to a multidisciplinary collaboration in which healthcare professionals (HCP) implement a series of evidence‐based perioperative management measures aiming to reduce traumatic stress reaction and promote the recovery of patients (Merchea& Larson, 2018). At present, ERAS has been carried out in fields including orthopaedic surgery, breast surgery, cardiothoracic surgery, gastrointestinal surgery, obstetrics and gynaecology, and has achieved remarkable results in reducing surgical stress response and perioperative complications, shortening the average length of hospital stay and reducing hospitalization costs (Asklid et al., 2017; Geubbels et al., 2019; Liu et al., 2017). The quality of enhanced recovery is very important for the prognosis, recovery speed and safety of patients (Asklid et al., 2017; Geubbels et al., 2019). However, quality of the implementation of ERAS is not optimistic, implementation of accelerated rehabilitation in practice is slow, and many clinical teams have not even incorporated the accelerated rehabilitation path into clinical practice (Elhassan et al., 2018; Francis et al., 2018). Studies have shown that during the implementation of ERAS, some medical staffs were not willing to implement accelerated rehabilitation measures, and the opposing colleagues was almost 68%, which needs to be improved (Martin et al., 2018). Keil et al. (2019) found that the compliance rate of the entire ERAS pathway was only about 58%. Nikodemski et al. (2017) found that patients with ERAS protocol did not require routine mechanical bowel preparation, but nearly 85% of patients received mechanical bowel preparation. Lambaudie et al. (2017) noted that only 69% of the HCP give supplement beverages containing sugar 2 h before surgery, and the early feeding was also needed to be improved. At present, it is not clear what difficulties medical staff have in implementing accelerated rehabilitation and what factors affect the effectiveness of implementing accelerated rehabilitation. Only by determining the factors affecting quality of accelerated rehabilitation can we promote the effective implementation of accelerated rehabilitation in clinical practice.

Patients with cervical spinal cord injury have severe trauma, high mortality, poor tolerance to surgery and great difficulty in perioperative nursing (Whelan et al., 2020). Early implementation of accelerated rehabilitation for patients with cervical spinal cord injury is very important to shorten the length of hospital stay, reduce hospitalization costs and promote quality rehabilitation for patients (Elsarrag et al., 2019). It is very important to monitor the nursing quality of accelerated rehabilitation for patients with cervical spinal cord injury, regulate behaviour of nursing staff implementing ERAS, ensure the quality of implementation of accelerated rehabilitation measures and promote rapid recovery of patients (Francis et al., 2018). At present, there is a lack of clinical practice guidelines or expert consensus on perioperative accelerated rehabilitation for patients with cervical spinal cord injury, and the quality of accelerated rehabilitation varies greatly.

## 2 BACKGROUND

In recent years, some scholars have actively explored the promotion and hindrance factors of enhanced recovery. Pędziwiatr et al. (2018) discussed the factors affecting the implementation of enhanced recovery, and results showed that lack of human resources, insufficient communication and collaboration of multidisciplinary teams and patients' resistance to change were the main reasons hindering the promotion of enhanced recovery. Gramlich et al. (2020) adopted qualitative interview method to investigate causes for enhanced recovery implementation difficulties. Results showed that the causes vary, including the time‐consuming nature of enhanced recovery measures, multidisciplinary collaboration difficulties, lack of medical support system and poor compliance of patients. Martin et al. (2018) interviewed the medical staff involved in enhanced recovery and discussed that the most important factors hindering the promotion of enhanced recovery were time restraints, insufficient support from ERAS care providers and insufficient logistical support. Meyenfeldt et al. (2022) used qualitative interview to discuss the reasons for difficulties in promoting enhanced recovery. The study showed that the main factors affecting enhanced recovery were poor compliance of staff, poor communication among members of multidisciplinary team, complexity of patients. At present, factors affecting quality of enhanced recovery are not completely clear. Therefore, it is difficult to improve the quality of enhanced recovery from a system level.

American scholar Donabedian proposed the structure‐process‐result theoretical model (Donabedian, 1992). Donabedian pointed out that the structural quality evaluation focused on the evaluation of basic working conditions for nursing work provided by the hospital. The process quality evaluation focused on the feedforward control of implementing medical procedure or treatment. Result quality evaluation was the objectified and datalized reflection of the practice outcome, belonging to the feedback control considered from patients' perspective (Denadai & Lo, 2021). Structure quality, process quality and result quality are closely related and linearly correlated in medical care services. A sound structure can improve process quality, a good process will have important impact on the quality of results, and the evaluation of results can also feedback and control the process of medical care, thus promoting the further improvement of medical care quality (Tossaint‐Schoenmakers et al., 2021). Accelerated rehabilitation quality evaluation is an important method to evaluate its effectiveness. It can evaluate the quality of accelerated rehabilitation, also can guide medical staff to standardize the accelerated rehabilitation work. Thus, it will ensure quality implementation of accelerated rehabilitation work (Tossaint‐Schoenmakers et al., 2021).

## 3 THE STUDY

### 3.1 Design

**FIGURE 1:**
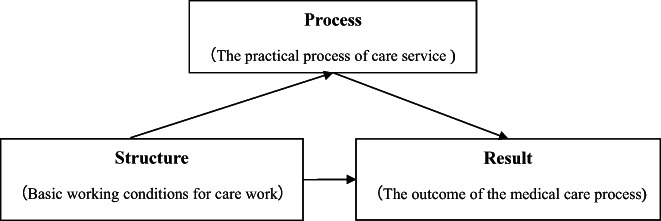
Three‐dimensional mass structure model (Donabedian, 1992).

This study employed a qualitative method with a descriptive and explorative design (Polit & Beck, 2010). Participants were invited to share and reflect on factors affecting quality of ERAS for patients with cervical spinal cord injury. Donabedian's structure‐process‐outcome theoretical model served as the theoretical framework of this study, as shown in Figure 1 (Donabedian, 1992). Descriptive qualitative research methods are considered useful for obtaining richer descriptions and are more suitable for exploring the factors affecting the quality of accelerated rehabilitation for patients with cervical spinal cord injury by medical staff (Luciani et al., 2019). Consolidated Criteria for Reporting Qualitative Research (COREQ) checklist for qualitative studies (Du et al., 2022) was adopted to report this study.

### 3.2 Participants and setting

From December 2020 to April 2021, the method of destination sampling was adopted and the principle of difference maximization was followed (Slamet et al., 2022). Medical staff from three tertiary hospitals in Beijing (Peking University International Hospital, Friendship Hospital Affiliated to Capital Medical University and Peking University Shougang Hospital) and one tertiary hospital in Sanya (Hainan Branch of PLA General Hospital) were selected for interviews. Among them, 14 medical staff in Beijing were interviewed face‐to‐face in the meeting room of the department of Orthopaedics. Two medical staff in Sanya City were interviewed online with Tencent Conference.

The inclusion criteria for participants are:

- Orthopaedic nurses, orthopaedic surgeons, orthopaedic head nurses, anaesthesiologists and physiotherapists with at least 1 year of practical experience in accelerated rehabilitation; More than 1 year of experience in accelerated rehabilitation ensures that participants have enough experience to comment on it.
- Willing to participate in this research.

We excluded medical staff who were taking a refresher course, on leave or away from clinical duties during the interview period.

The sample size of the interview is based on the principle of data saturation, that is, data collection will stop when there is no more new information or content (Hennink & Kaiser, 2022). A total of 16 interviewees were interviewed in this study, including eight orthopaedic nurses and head nurses, four orthopaedic surgeons, two physiotherapists and two anaesthesiologists. Among them, nine were females and seven were males. Four of the interviewees have a PhD degree and the rest have a bachelor degree. Respondents ranged in age from 29 to 51 years old. Working experience in spinal surgery ranged from 6 to 32 years. The practical experience of accelerated rehabilitation was 4–6 years. A total of five supplementary interviews were conducted with four respondents. General information about respondents is shown in Table 1.

**TABLE 1:** General information of respondents (*N* = 16).

Number	Gender	Age (years)	Job	Education	Years of experience in spinal surgical department	Years of ERAS practice experience
N1	Female	32	Orthopaedic nurse	Bachelor	10	4
N2	Female	39	Orthopaedic nurse	Bachelor	16	4
N3	Female	41	Head nurse	Bachelor	19	6
N4	Female	38	Orthopaedic nurse	Bachelor	19	4
N5	Male	32	Orthopaedic nurse	Bachelor	11	4
N6	Female	40	Head nurse	Bachelor	19	4
N7	Female	43	Orthopaedic nurse	Bachelor	25	6
N8	Male	34	Orthopaedist	Doctor	10	4
N9	Male	35	Orthopaedist	Doctor	10	4
N10	Male	35	Orthopaedist	Doctor	11	4
N11	Female	51	Orthopaedic nurse	Bachelor	32	6
N12	Male	35	Orthopaedist	Doctor	11	4
N13	Female	32	Physical therapist	Bachelor	10	4
N14	Female	29	Physical therapist	Bachelor	6	4
N15	Male	33	Anaesthetist	Bachelor	9	4
N16	Male	36	Anaesthetist	Bachelor	11	4

### 3.3 Data collection

The data were collected in the form of face‐to‐face semi‐structured interviews. The interview outline was preliminarily formulated according to the research purpose and Donabedian's structure‐process‐result theoretical model (Donabedian, 1992). The interview outline was determined based on the pre‐interview of two nurses with accelerated rehabilitation experience. The final interview outline was as follows: (1) What difficulties did you encounter in the process of applying accelerated rehabilitation for patients with cervical spinal cord injury? (2) Before applying accelerated rehabilitation, what factors do you think affect the quality of accelerated rehabilitation? (3) In the process of accelerated rehabilitation, what factors do you think affect its quality?

Some interviewees were colleagues, and some were introduced by colleagues. Before the interview, time and place of interview were confirmed with the interviewees, and the time period convenient for interviewees were chosen. The interviews took place in a conference room of the hospital ward. Interviews were conducted in a quiet environment, with only the interviewer and interviewee present. Interviewers had systematically learned interview methods and skills before the formal interviews, and a pre‐interview was conducted. After each interview, the author kept a reflection diary to reflect on the existing problems and correct them in the upcoming interviews. Interviews were conducted following the interview outline. During interviews, interviewees were carefully listened to and properly responded to, so that they could fully express their views. Key information of interviews was recorded in time, and non‐verbal behaviours such as verbal pauses, facial expressions and actions of interviewees were closely observed and recorded. Researcher maintained a non‐biased attitude to maintain objectivity of data collection. After interviews, gratitude for participation was expressed and possibility of follow up encounters was explained in case of further addition of information. Each interview lasted for 30–45 min.

### 3.4 Data analysis

The interview recording was transcribed into text within 24 h after the interview. Original interview materials were read repeatedly, reviewed with interviewees for verification and consent for application. The data were analysed by theme analysis method (Kiger & Varpio, 2020). The steps were as follows, (1) Repeatedly read the transcript take notes. (2) Extract duplicated points in the data and encode them with phrases or sentences. (3) Further condense the codes with similar contents to generate themes/sub‐themes. (4) Review the themes/subthemes and compare it with the original data repeatedly. (5) Rephrase the themes in simple and easily‐understandable words. (6) Write data analysis process. To reduce the subjectivity of the study, two researchers analysed the data independently and held regular discussions until the encoded information was agreed upon.

### 3.5 Rigour and trustworthiness

#### 3.5.1 Credibility

To ensure the credibility of results, data were returned to interviewees for verification after transcription into text. Two researchers reviewed the data repeatedly and independently, and discussed results of data analysis with other members of the research group on a regular basis about inconsistencies in the coding. In case of disagreement, the original data were reviewed immediately with comparison with field notes, reflective diaries and other data to truly reflect the data content from multiple perspectives and improve the credibility of the data. And the decision was made by the third researcher (Cypress, 2017).

#### 3.5.2 Transferability

According to the principle of difference maximization (Chen, 2000), targeted multidisciplinary HCP with accelerated rehabilitation experience in cervical and spinal cord injury were selected for interviews, to ensure the diversity of participant source. (Chen, 2000).

#### 3.5.3 Confirmability

In order to ensure the verifiability of this study, entire process of the interview was recorded, so that researchers could check the record at any time (Thompson Burdine et al., 2021).

#### 3.5.4 Dependability

Inclusion and exclusion criteria, data collection methods and data analysis methods were described in detail (Luciani et al., 2019).

### 3.6 Ethical consideration

The study was performed following the Helsinki principles of Ethics (Kurihara, et al., 2020). The study was approved by the Ethics Committee of Capital Medical University (batch number: Z2022SY032). Approval date is March 9, 2022. The aim and significance of the research were fully informed to interviewees before the interview. We have also promised to keep the relevant information strictly confidential. Information was to be shared within the research group only. Respondents were told that they could voluntarily participate in this study and were able to withdraw from this study at any time. During the interview, all interviewees participated voluntarily, without any refusal or withdrawal. Each participant has received some compensation for their time contributed (like towels, thermos cups, etc.).

## 4 FINDINGS

A total of two themes and nine sub‐themes were extracted in this study, among which three sub‐themes were related to the structural quality of accelerated rehabilitation and six sub‐themes were related to its process quality.

### 4.1 Theme 1: Factors related to the structure quality of ERAS

#### 4.1.1 Build multidisciplinary teams

Some participants emphasized the importance of building an accelerated rehabilitation team to improve quality. This requires multidisciplinary HCP including orthopaedic surgeons, physicians, orthopaedic nurses, anaesthesiologists, operating room nurses, physiotherapists, and dieticians to perform their respective roles and collaborate effectively.

It requires multidisciplinary, physician‐led collaboration, including the anesthesiologists performing the preoperative evaluation, and the orthopedic surgeons completing the preoperative examination and assessment. Physicians could evaluate conditions such as hypertension, diabetes, cardiac disease, etc. There should be nutrition doctors to evaluate the nutritional status of patients and provide nutrition guidance and so on. (P7)

Also, some participants emphasized that nurses play an important role in the accelerated rehabilitation team and have a direct impact on the quality of accelerated rehabilitation. In addition, patient participation plays a crucial role in improving the quality of rehabilitation.

It is important for patients to be actively involved. Nurses play an important role in the evaluation and early identification of rehabilitation and postoperative complications, which is directly related to the overall nursing quality of patients, and is also the inspector of the patients' knowledge of rehabilitation. (P2)

#### 4.1.2 Complete system guarantee

Some participants expressed that only by establishing standard guidelines, policies for clinical pathways related to accelerate rehabilitation of cervical and spinal cord injuries can the behaviours be regulated. The implementation of accelerated rehabilitation work can therefore be better guided and the homogeneous diagnosis and treatment be achieved.

It is very necessary for us to establish a comprehensive rule and regulation, so as to better regulate the behaviors of the members of accelerated rehabilitation team and facilitate the inspection of the work standards of the HCP. (P1)

There should be a clinical pathway for diagnosis and treatment of cervical spinal cord injury. I think the establishment of this standard is very critical, which mainly includes regulations, process, nursing routine, medication routine and follow‐up rehabilitation guidance routine. Only by establishing standardized diagnosis and treatment norms can we carry out homogenous diagnosis and treatment in a systematic and process‐based way. (P6)

#### 4.1.3 Adequate staffing

Allocation of human resources is considered to be a structural factor affecting quality of accelerated rehabilitation. Adequate resources are the prerequisite. Some participants expressed that due to the implementation of accelerated rehabilitation, workload of nurses has increased, resulting in a relative shortage of human resources, which was a hindrance to the implementation of accelerated rehabilitation measures by nursing staff.

For patients who can urinate spontaneously, the urinary catheter should be removed on the day after surgery according to idea of enhanced recovery. However, sometimes when patients return to the ward after surgery, it is during night shift, with less staffing and larger workload. If the patient cannot urinate (cannot pass trial of void) after the catheter has been removed, the catheter must be re‐inserted. Therefore, the nurse usually removes the catheter during day shift the next morning, so the enhanced recovery cannot be implemented due to the shortage of nurses. (P3)

The workload of nurses is too heavy now, and there is no time to supervise whether patients are doing rehabilitation exercises properly, such as training time and standard movements. (P6)

### 4.2 Theme 2: Factors related to the process quality of ERAS

#### 4.2.1 Inadequate training and assessment

Medical staff with necessary knowledge of accelerated rehabilitation is the premise. Inadequate training of HCP was identified as an important factor affecting the quality of accelerated rehabilitation process. Some participants expressed that medical staff's awareness of accelerated rehabilitation would directly affect the accuracy, timeliness and effect of implementing accelerated rehabilitation measures.

I think both the awareness and implementation process of enhanced recovery need to be relearned. Due to the different comprehension ability of each nurse, the implementation effect of enhanced recovery is different. Therefore, managers need to train nurses repeatedly, so that all people can really master the enhanced recovery and specific implementation methods. The leaders of our department usually send some key nurses to participate in the training of enhanced recovery on a regular basis, but this kind of training only enables a small number of people in the department to understand the relevant knowledge of enhanced recovery. In my opinion, in order to truly promote the application of enhanced recovery, all nurses should be trained. Only with full understanding of enhanced recovery can we actively implement nursing measures. (P4)

In addition, assessment of HCP' knowledge of accelerated rehabilitation is considered to be an important factor to ensure the quality of accelerated rehabilitation, so as to promote HCP' awareness of accelerated rehabilitation and ensure the provision of homogeneous care for patients.

ERAS is a new concept, and it is difficult for medical staff to thoroughly understand the importance of enhanced recovery. Therefore, training on enhanced recovery should be strengthened and courses related to enhanced recovery should be set up to improve team effectiveness. (P13)

#### 4.2.2 Awareness of medical staff

Recognition of the importance and the expected benefits of ERAS by HCP are considered to be contributing factors. Some participants said that some medical staff had outdated concepts and could not realize the importance of accelerated rehabilitation, which would affect its promotion and effect.

In the process of promoting enhanced recovery, some medical staff have outdated cognition, takes a long time to accept new concepts, and are skeptical about the concept of enhanced recovery. In addition, the short‐term benefits of enhanced recovery are not obvious, so that some medical staff still take conventional nursing measures, delaying the promotion of accelerated rehabilitation. (P2)

In addition, some participants indicated that medical staff's recognition of the benefits of accelerated rehabilitation during practice could strengthen their awareness of its importance.

At the beginning, people didn't know much about enhanced recovery, but our medical team saw the benefits of enhanced recovery to patients through the implementation of enhanced recovery measures in the past three years, and constantly increased our understanding of enhanced recovery. (P6)

#### 4.2.3 Members of the accelerated rehabilitation team are inadequate

The lack of capacity of accelerated rehabilitation HCP is considered to be a factor affecting the process quality of the accelerated rehabilitation. Some interviewees indicated that the lack of HCP's ability would affect the implementation of accelerated rehabilitation programs for patients and the development of personalized accelerated rehabilitation clinical pathways, thus affecting the quality of accelerated rehabilitation.

The competence and experience of the nurses in the team will influence the implementation of the accelerated rehabilitation program. Firstly, it needs to be above average (P12).

In the process of postoperative rehabilitation for patients, nurses should not implement the same rehabilitation plan for every patient, but should timely adjust the rehabilitation plan based on the accelerated rehabilitation process and according to different patients' conditions and tolerance degrees (P8).

#### 4.2.4 Poor multidisciplinary communication and collaboration

In the process of implementing accelerated rehabilitation, it is necessary for multidisciplinary HCP to reach a consensus on the requirements of implementation of accelerated rehabilitation, so as to carry out homogenized management for patients. Some participants mentioned that providing patients with adequate and consistent information is an important factor affecting the implementation of accelerated rehabilitation. Good multidisciplinary communication can promote the implementation of accelerated rehabilitation, but inconsistent information or poor communication is an obstacle to the effective operation of accelerated rehabilitation.

Enhanced recovery cannot be completed by one department. It requires the cooperation of multiple departments. There may be some problems in the process of cooperation. The anesthesiology nurse said that the patient could only drink water six hours after returning from the operating room, but we taught the patient two hours. (P1)

Generally, first day after surgery involves mainly bed function exercise, on second day patients can wear braces, sit up by the bed, encourage patients to get out of bed for early activities if no symptoms of dizziness or limb weakness. At this time reducing unnecessary infusion is required, as well as increasing the time spent out of bed by patients, but it is necessary to communicate with the doctor in charge. (P2)

#### 4.2.5 The patient's cognitive deficit

Patients' compliance to accelerated rehabilitation program is an important factor affecting the quality and effectiveness of accelerated rehabilitation. Some participants indicated that due to the influence of traditional concepts, some patients are not aware of the benefits and safety of accelerated rehabilitation. They tend to adopt a conservative attitude towards accelerated rehabilitation and cannot strictly implement the measures related to accelerated rehabilitation, thus affecting patients' compliance.

Enhanced recovery requires patient's cooperation, but due to the patients' fixed thinking in the past, they may not believe in the concept of enhanced recovery. Some patients are conservative and do not accept enhanced recovery. (P1)

Some patients believe muscle and bone injuries takes about 100 days to recover. They believe that they should remain in bed to reduce physical activity during recovery. Therefore, the compliance is very poor when we tell patients to do rehabilitation exercise, and it is very difficult to complete the enhanced recovery program. (P14)

#### 4.2.6 Effective health education

Health education is considered to be an important factor affecting the quality of accelerated rehabilitation. Some participants indicated that education for patients and family members about the benefits of accelerated rehabilitation could improve their awareness of the benefits and thus patient compliance. Therefore, the quality of accelerated rehabilitation can be enhanced.

We should begin to teach patients regarding enhanced recovery from admission. We should let patients and their family know what is enhanced recovery, what is good for them, and make them fully understand about the benefits. It then might be easier for us to carry out the work. (P6)

Most patients do not know much about enhanced recovery, so the whole team needs to carry out standardized propaganda and education for patients. After repeated propaganda and education, patients will form a positive perception of enhanced recovery. If propaganda and education are not in place, patients will not cooperate actively, and the rehabilitation effect of patients will be poor. (P14)

### 4.3 The conceptual framework influencing the quality of enhanced recovery

**FIGURE 2:**
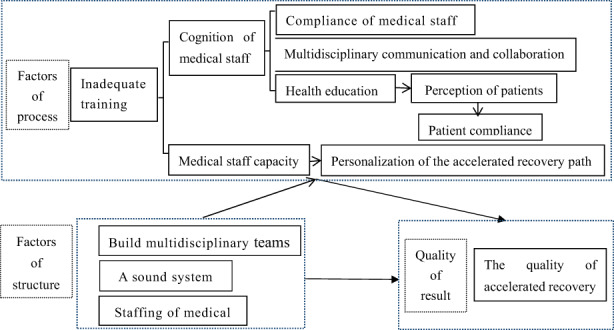
Conceptual framework influencing the quality of accelerated recovery.

Through the analysis of interview data, we extracted the conceptual framework affecting the quality of accelerated rehabilitation, as shown in Figure 2. Among them, the lack of nursing staff is structural factor, and the lack of training of accelerated rehabilitation members is process factor. Lack of training affects the awareness of HCP of accelerated rehabilitation, hence affects the ability, compliance and multidisciplinary collaboration between HCP. In addition, HCP' perceptions also affect patients' perceptions about accelerated rehabilitation, thus affecting patients' compliance and quality of accelerated rehabilitation.

## 5 DISCUSSION

In this study, the qualitative descriptive research method was adopted to explore the awareness of medical staff on the factors affecting the quality of accelerated rehabilitation for patients with cervical spinal cord injury. It can provide reference for promoting the quality of nursing care. Through the analysis of interview results, we extracted factors affecting quality of accelerated rehabilitation for patients with cervical spinal cord injury and grouped into two themes. Among them, factors related to quality of accelerated rehabilitation structure have three sub‐themes, including the construction of multidisciplinary team, complete system guarantee, and adequate staffing. Factors related to quality of accelerated rehabilitation process have six sub‐themes, including inadequate training of medical staff, backward concept of medical staff, incapability of members of accelerated rehabilitation team, difficulty in collaboration among multidisciplinary members, lack of awareness of patients and lack of adequate health education for patients. The results of this study were consistent with the framework of structure‐process‐outcome theoretical model (Donabedian, 1992). It extended the application of this theoretical model in the field of quality management of accelerated rehabilitation for patients with cervical spinal cord injury. The research results can provide reference for future design of accelerated rehabilitation quality improvement projects for patients with cervical spinal cord injury. Some results of this study are consistent with those of previous qualitative studies (Gramlich et al., 2017). Our study emphasized the importance of training and regular communication with members of accelerated rehabilitation team, so that the HCP can maintain consistent understanding of the importance, benefits and implementation plan of accelerated rehabilitation.

### 5.1 Factors related to structural quality of accelerated rehabilitation

In this study, participants reported factors related to structural quality of accelerated rehabilitation, includes construction of multidisciplinary teams, establishment of thorough systems and adequate staffing. Previous studies have emphasized the importance of establishing accelerated rehabilitation teams and their effective operation. In the process of accelerated rehabilitation, multidisciplinary team service model shows the advantage of collaboration, it can make full use of existing resources, provide patients with more comprehensive diagnosis and treatment services, and improve patient satisfaction (Medbery et al., 2019). Rossettini et al. (2021) believed that only when HCP truly realize their role in the team can they improve their level of performance and promote implementation of accelerated rehabilitation. Previous studies have also pointed out that the establishment of a complete accelerated rehabilitation system can better standardize the implementation of accelerated rehabilitation measures by medical staff, hence providing patients with standard, unified and standardized diagnosis and treatment services, and improving the implementation rate and efficiency of accelerated rehabilitation. Therefore, it ensures the orderly operation of accelerated rehabilitation team, and helps realize standardized clinical management (Long‐Jun et al., 2018; Smirk et al., 2018; Sun et al., 2019). This study highlights the importance of adequate nursing staff before intensive rehabilitation. Nurses are the main implementers of accelerated rehabilitation. They need to develop and implement accelerated rehabilitation nursing program, and evaluate the therapeutic effect. They play an extremely important role in the accelerated rehabilitation multidisciplinary team (Awad et al., 2019; Ljungqvist et al., 2017). Therefore, if the allocation of nursing resources is insufficient, it will affect the implementation and quality of accelerated rehabilitation.

Training of accelerated rehabilitation HCP is an important factor affecting quality of accelerated rehabilitation process. This was consistent with previous studies (Gramlich et al., 2017). The knowledge of accelerated rehabilitation HCP will directly affect their awareness of accelerated rehabilitation, hence affecting their compliance of implementation (Francis et al., 2018; Spinal cord Injury Prevention and Control Group et al., 2019). Finally, it can impact the effectiveness of implementation of accelerating rehabilitation (Surgical Society of Chinese Medical Association & Chinese Society of Anesthesiology, 2018; Aliza et al., 2017). It takes a long time for the concept of accelerated rehabilitation to be widely applied in clinical practice. Medical staff have been affected by traditional concepts for a long time, and their concept is difficult to be altered completely in a short period of time. This affects the implementation of specific measures of accelerate rehabilitation (Gramlich et al., 2020). In addition, due to the contradiction between some accelerated rehabilitation and traditional measures, medical staff did not immediately see the outcome of accelerated rehabilitation, and they were sceptical about the effect of accelerated rehabilitation. Hence, medical staff were not ready to accept the accelerated rehabilitation program. As a result, the implementation of accelerated rehabilitation measures is poor.

Difficulties in communication and collaboration among multidisciplinary members are also important factors affecting the quality of accelerated rehabilitation. This is consistent with previous research results (Gramlich et al., 2017). The timeliness and accuracy of information communicated among multidisciplinary HCP is the key to promote the smooth implementation of accelerated rehabilitation (Rossettini et al., 2021). Strengthening communication and coordination between medical and nursing staff is conducive to promote accelerated rehabilitation (Brown & Xhaja, 2018; Crosson, 2018; Sibbern et al., 2017). Awad et al. (2019) also believed that face‐to‐face communication among multidisciplinary HCP should be strengthened to facilitate better inter‐disciplinary cooperation. Brown and Xhaja (2018) showed that lack of communication among multidisciplinary HCP is one of the factors affecting implementation of ERAS. This study emphasizes that the cognitive difference of medical staff on accelerated rehabilitation is one of the reasons for poor communication among multidisciplinary members during implementation of accelerated rehabilitation. Therefore, consistent training for accelerated rehabilitation HCP is critical.

The lack of adequate health education and lack of awareness of accelerated rehabilitation are important factors affecting the process quality of accelerated rehabilitation. This is consistent with previous studies (Carmichael et al., 2017; Gramlich et al., 2017). Due to deep‐rooted traditional concepts and uneven educational levels of patients, some patients do not understand accelerated rehabilitation mode and have doubts about it, leading to their preference for conservative management. Health education is an important way for patients and their families to acquire disease‐related knowledge, and directly affects patients' acceptance of accelerated rehabilitation program, psychological state and rehabilitation effect (Inkeroinen et al., 2020). Carmichael et al. (2017) believed that effective health education on accelerated rehabilitation could improve patients' compliance. Only when patients fully realize the importance of accelerated rehabilitation, can they accept this concept, hence actively cooperate with medical staff to implement rehabilitation programs and improve the effect of accelerated rehabilitation.

The lack of competency of the accelerated rehabilitation HCP is also a factor affecting the quality of accelerated rehabilitation. When the competency of the nurse is low, it will be difficult to personalize the clinical path to ERAS for the patient (Wang et al., 2022). The general clinical pathway of accelerated rehabilitation cannot meet the individual needs for each patient. The establishment of personalized clinical pathways can help medical staff clarify the specific content and process of implementation of accelerated rehabilitation for individual patient. Therefore, implementation of accelerated rehabilitation measures will be better regulated by medical staff. (Smirk et al., 2018). Smirk et al. (2018) believe that due to the variability in each patient's condition, multidisciplinary team should develop personalized and targeted diagnosis and treatment plan for each patient, so that each patient can receive targeted diagnosis and treatment. This shortens the rehabilitation period for patients, and makes diagnosis and treatment and nursing work more efficient.

## 6 STRENGTHS AND LIMITATIONS OF THE RESEARCH

In this study, Donabedian's structure‐process‐result theory model is used as the framework to refine the themes, making the extracted themes more scientific (Donabedian, 1996). The qualitative interview method was used to select orthopaedic nurses, nursing management experts, orthopaedic doctors, anaesthesiologists and physiotherapists of different ages, genders and positions with accelerated rehabilitation experience, reveal their different views on the impact of accelerated rehabilitation quality, and obtain some valuable opinions, which can provide reference for improving the quality of accelerated rehabilitation implementation. This study clearly and comprehensively describes the research process, thus ensuring the validity of the study. The initial citation explains the process of analysis and drawing conclusions from the results, which increases the credibility of the research results.

However, this study also has some limitations. First of all, this study only discussed the views of medical staff on the factors affecting the quality of accelerated rehabilitation, and did not discuss the views of patients. Secondly, although the data in this study reached saturation, interviewees only came from two cities in China. It may limit the universality of the research results. In addition, most of interviews were conducted face‐to‐face, but interviewees were all wearing masks due to the epidemic. Thus, it was impossible to observe the facial expressions of the interviewees and record their reactions in detail. Online video interview was conducted with two interviewees, and interview effect may be lower than that in real environment.

## 7 CONCLUSIONS AND RELEVANCE TO CLINICAL PRACTICE

This study provided a new insight into the factors influencing the quality of accelerated rehabilitation, and could provide a reference for managers to develop the quality improvement program of accelerated rehabilitation for patients with cervical spinal cord injury during perioperative period, and constructed the evaluation index system for nursing quality.

This study emphasized that before the implementation of accelerated rehabilitation, hospital managers should be equipped with sufficient nursing resources and should allocate nursing resource reasonably according to the workload during implementation of accelerated rehabilitation. At the same time, manager should establish a system related to accelerated rehabilitation and organize training and assessment regularly. This is to ensure HCP to achieve unity of the concept of accelerated rehabilitation. In addition, managers should strengthen leadership, organize HCP to discuss the problems encountered in the work regularly, promote communication and collaboration of accelerated rehabilitation members, in order to reach a consensus on the specific measures of accelerated rehabilitation, and ensure the quality of implementing accelerated rehabilitation.

For HCP, they should actively learn the knowledge of accelerated rehabilitation, improve their own capability, and develop personalized clinical pathways for patients based on their conditions. Secondly, in the process of implementing accelerated rehabilitation, HCP should take the initiative to communicate with other members in order to timely solve the problems in the process of accelerated rehabilitation and reach an agreement. In addition, HCP should learn the up‐to‐date guidelines for accelerated rehabilitation on regular basis. They should reflect on the implementation of accelerated rehabilitation to continuously improve the quality.

## AUTHOR CONTRIBUTIONS

Interview, data collection, data analysis and writing— original draft preparation: Qiuxue Li; Data collection, data analysis, proofreading: Qianghuizi Zhang. Design, supervision and review the entire project: Weiwei Liu; the revised version of the manuscript, polish the language: Zheyi Zhou. Discuss the results, provide feedback, Acknowledgement and agreement with the content of the article: All authors.

## CONFLICT OF INTEREST STATEMENT

The authors declare that they have no conflict of interests.

## FUNDING INFORMATION

There is no funding for this article.

## RESEARCH ETHICS COMMITTEE APPROVAL

Capital Medical University (batch number: Z2022SY032).The purpose and significance of the research were fully informed to the interviewees before the interview.

## Data Availability

The data that support the findings of this study are available from the corresponding author upon reasonable request.

## References

[nop21715-bib-0002] Asklid, D. , Segelman, J. , Gedda, C. , Hjern, F. , Pekkari, K. , & Gustafsson, U. O. (2017). The impact of perioperative fluid therapy on short‐term outcomes and 5‐year survival among patients undergoing colorectal cancer surgery – a prospective cohort study within an ERAS protocol. European Journal of Surgical Oncology, 43(8), 1433–1439. 10.1016/j.ejso.2017.04.003 28528188

[nop21715-bib-0003] Awad, F. , Searle, D. , Walmsley, K. , Dyar, N. , Auckland, C. , Bethune, R. , Eyres, K. , Toms, A. D. , & Phillips, J. (2019). The Exeter knee infection multi disciplinary team approach to managing prosthetic knee infections: A qualitative analysis. Journal of Orthopaedics, 18, 86–90. 10.1016/j.jor.2019.09.004 32189890 PMC7068000

[nop21715-bib-0004] Brown, D. , & Xhaja, A. (2018). Nursing perspectives on enhanced recovery after surgery. The Surgical Clinics of North America, 98(6), 1211–1221. 10.1016/j.suc.2018.07.008 30390853

[nop21715-bib-0005] Carmichael, J. C. , Keller, D. S. , Baldini, G. , Bordeianou, L. , Weiss, E. , Lee, L. , Boutros, M. , McClane, J. , Feldman, L. S. , & Steele, S. R. (2017). Clinical practice guidelines for enhanced recovery after colon and Rectal surgery from the American Society of Colon and Rectal Surgeons and Society of American Gastrointestinal and Endoscopic Surgeons. Diseases of the Colon and Rectum, 60(8), 761–784. 10.1097/DCR.0000000000000883 28682962

[nop21715-bib-0006] Chen, X. M. (2000). Qualitative research methods and social science research【M】 (pp. 5–7). Educational Science.

[nop21715-bib-0008] Crosson, J. A. (2018). Enhanced recovery after surgery‐the importance of the Perianesthesia nurse on program success. Journal of Perianesthesia Nursing, 33(4), 366–374. 10.1016/j.jopan.2016.09.010 30077278

[nop21715-bib-0009] Cypress, B. S. (2017). Rigor or reliability and validity in qualitative research: Perspectives, strategies, reconceptualization, and recommendations. Dimensions of Critical Care Nursing, 36(4), 253–263. 10.1097/DCC.0000000000000253 28570380

[nop21715-bib-0010] Denadai, R. , & Lo, L. J. (2021). Adapting Donabedian's structure‐process‐outcome triad for quality improvement activities in surgical cleft‐craniofacial care. Journal of Plastic, Reconstructive & Aesthetic Surgery, 74(1), 223–243. 10.1016/j.bjps.2020.05.073 32507708

[nop21715-bib-0011] Donabedian, A. (1992). Quality assurance. Structure, process and outcome. Nursing Standard (Royal College of Nursing (Great Britain): 1987), 7(11 Suppl QA), 4–5. 1489693

[nop21715-bib-0012] Donabedian, A. (1996). Evaluating the quality of medical care. The Milbank Quarterly, 44(3), 691–729. 10.1111/j.1468-0009.2005.00397.x PMC2690293 16279964

[nop21715-bib-0013] Du, K. J. , Li, G. S. , Zhang, K. , Lin, Y. , Yang, F. , & Hannes, K. (2022). Prof. Karin Hannes: COREQ (consolidated criteria for reporting qualitative studies). Annals of Translational Medicine, 10(19), 1073. 10.21037/atm-2022-23 36330400 PMC9622469

[nop21715-bib-0014] Elhassan, A. , Ahmed, A. , Awad, H. , Humeidan, M. , Nguyen, V. , Cornett, E. M. , Urman, R. D. , & Kaye, A. D. (2018). The evolution of surgical enhanced recovery pathways: A review. Current Pain and Headache Reports, 22(11), 74. 10.1007/s11916-018-0727-z 30171357

[nop21715-bib-0015] Elsarrag, M. , Soldozy, S. , Patel, P. , Norat, P. , Sokolowski, J. D. , Park, M. S. , Tvrdik, P. , & Kalani, M. Y. S. (2019). Enhanced recovery after spine surgery: A systematic review. Neurosurgical Focus, 46(4), E3. 10.3171/2019.1.FOCUS18700 30933920

[nop21715-bib-0016] Francis, N. K. , Walker, T. , Carter, F. , Hübner, M. , Balfour, A. , Jakobsen, D. H. , Burch, J. , Wasylak, T. , Demartines, N. , Lobo, D. N. , Addor, V. , & Ljungqvist, O. (2018). Consensus on training and implementation of enhanced recovery after surgery: A Delphi study. World Journal of Surgery, 42(7), 1919–1928. 10.1007/s00268-017-4436-2 29302724

[nop21715-bib-0017] Geubbels, N. , Evren, I. , Acherman, Y. I. Z. , Bruin, S. C. , van de Laar, A. W. J. M. , Hoen, M. B. , & de Brauw, L. M. (2019). Randomized clinical trial of an enhanced recovery after surgery programme versus conventional care in laparoscopic roux‐en‐Y gastric bypass surgery. BJS Open, 3(3), 274–281. 10.1002/bjs5.50143 31183442 PMC6551390

[nop21715-bib-0018] Gramlich, L. , Nelson, G. , Nelson, A. , Lagendyk, L. , Gilmour, L. E. , & Wasylak, T. (2020). Moving enhanced recovery after surgery from implementation to sustainability across a health system: A qualitative assessment of leadership perspectives. BMC Health Services Research, 20(1), 361. 10.1186/s12913-020-05227-0 32336268 PMC7183608

[nop21715-bib-0019] Gramlich, L. M. , Sheppard, C. E. , Wasylak, T. , Gilmour, L. E. , Ljungqvist, O. , Basualdo‐Hammond, C. , & Nelson, G. (2017). Implementation of enhanced recovery after surgery: A strategy to transform surgical care across a health system. Implementation Science, 12(1), 67. 10.1186/s13012-017-0597-5 28526041 PMC5438526

[nop21715-bib-0020] Hennink, M. , & Kaiser, B. N. (2022). Sample sizes for saturation in qualitative research: A systematic review of empirical tests. Social Science & Medicine, 292(1982), 114523. 10.1016/j.socscimed.2021.114523 34785096

[nop21715-bib-0021] Inkeroinen, S. , Virtanen, H. , Kilpi, T. , Laulaja, J. , Puukka, P. , Tuominen, R. , & Leino‐Kilpi, H. (2020). Relationship between sufficiency and usefulness of patient education: A cross‐sectional study of patients with chronic kidney disease. Nursing & Health Sciences, 22(4), 846–853. 10.1111/nhs.12770 32840003

[nop21715-bib-0022] Keil, D. S. , Schiff, L. D. , Carey, E. T. , Moulder, J. K. , Goetzinger, A. M. , Patidar, S. M. , Hance, L. M. , Kolarczyk, L. M. , Isaak, R. S. , Strassle, P. D. , & Schoenherr, J. W. (2019). Predictors of admission after the implementation of an enhanced recovery after surgery pathway for minimally invasive gynecologic surgery. Anesthesia and Analgesia, 129(3), 776–783. 10.1213/ANE.0000000000003339 31425219

[nop21715-bib-0023] Kiger, M. E. , & Varpio, L. (2020). Thematic analysis of qualitative data: AMEE guide No. 131. Medical Teacher, 42(8), 846–854. 10.1080/0142159X.2020.1755030 32356468

[nop21715-bib-0024] Kurihara, C. , Baroutsou, V. , Becker, S. , Brun, J. , Franke‐Bray, B. , Carlesi, R. , Chan, A. , Collia, L. F. , Kleist, P. , Laranjeira, L. F. , Matsuyama, K. , Naseem, S. , Schenk, J. , Silva, H. , Kerpel‐Fronius, S. , & Working Group on Ethics of the International Federation of Associations of Pharmaceutical Physicians and Pharmaceutical Medicine . (2020). Linking the declarations of Helsinki and of Taipei: Critical challenges of future‐oriented research Ethics. Frontiers in Pharmacology, 11, 579714. 10.3389/fphar.2020.579714 33324212 PMC7723451

[nop21715-bib-0025] Lambaudie, E. , de Nonneville, A. , Brun, C. , Laplane, C. , N'Guyen Duong, L. , Boher, J. M. , Jauffret, C. , Blache, G. , Knight, S. , Cini, E. , Houvenaeghel, G. , & Blache, J. L. (2017). Enhanced recovery after surgery program in Gynaecologic oncological surgery in a minimally invasive techniques expert center. BMC Surgery, 17(1), 136. 10.1186/s12893-017-0332-9 29282059 PMC5745717

[nop21715-bib-0026] Liu, V. X. , Rosas, E. , Hwang, J. C. , Cain, E. , Foss‐Durant, A. , Clopp, M. , Huang, M. , Mustille, A. , Reyes, V. M. , Paulson, S. S. , Caughey, M. , & Parodi, S. (2017). The Kaiser Permanente northern California enhanced recovery after surgery program: Design, development, and implementation. The Permanente Journal, 21, 17‐003. 10.7812/TPP/17-003 PMC5528846 28746028

[nop21715-bib-0027] Ljungqvist, O. , Scott, M. , & Fearon, K. C. (2017). Enhanced recovery after surgery: A review. JAMA Surgery, 152(3), 292–298. 10.1001/jamasurg.2016.4952 28097305

[nop21715-bib-0028] Long‐Jun, H. U. , Fei, H. X. , & Wang, Q. J. (2018). Practice of enhanced recovery after surgery based on theory of business process reengineering. Chinese Hospital Management, 38(12), 56–57.

[nop21715-bib-0029] Luciani, M. , Jack, S. M. , Campbell, K. , Orr, E. , Durepos, P. , Li, L. , Strachan, P. , & Di Mauro, S. (2019). An Introduction to qualitative Health Research. (Un'introduzione alla ricerca sanitaria qualitativa). Professioni Infermieristiche, 72(1), 60–68. 31162045

[nop21715-bib-0030] Martin, D. , Roulin, D. , Grass, F. , Addor, V. , Ljungqvist, O. , Demartines, N. , & Hübner, M. (2018). A multicentre qualitative study assessing implementation of an enhanced recovery after surgery program. Clinical Nutrition (Edinburgh, Scotland), 37(6 Pt A), 2172–2177. 10.1016/j.clnu.2017.10.017 29129637

[nop21715-bib-0031] Medbery, R. L. , Fernandez, F. G. , & Khullar, O. V. (2019). ERAS and patient reported outcomes in thoracic surgery: A review of current data. Journal of Thoracic Disease, 11(Suppl 7), S976–S986. 10.21037/jtd.2019.04.08 31183180 PMC6535471

[nop21715-bib-0032] Merchea, A. , & Larson, D. W. (2018). Enhanced recovery after surgery and future directions. The Surgical Clinics of North America, 98(6), 1287–1292. 10.1016/j.suc.2018.07.014 30390860

[nop21715-bib-0033] Meyenfeldt, E. M. V. , van Nassau, F. , de Betue, C. T. I. , Barberio, L. , Schreurs, W. H. , Marres, G. M. H. , Bonjer, H. J. , & Anema, J. (2022). Implementing an enhanced recovery after thoracic surgery programme in The Netherlands: A qualitative study investigating facilitators and barriers for implementation. BMJ Open, 12(1), e051513. 10.1136/bmjopen-2021-051513 PMC8734011 34987041

[nop21715-bib-0034] Nikodemski, T. , Biskup, A. , Taszarek, A. , Albin, M. , Chudecka‐Głaz, A. , Cymbaluk‐Płoska, A. , & Menkiszak, J. (2017). Implementation of an enhanced recovery after surgery (ERAS) protocol in a gynaecology department – the follow‐up at 1 year. Contemporary Oncology (Poznan, Poland), 21(3), 240–243. 10.5114/wo.2017.69589 29180933 PMC5701575

[nop21715-bib-0035] Pędziwiatr, M. , Mavrikis, J. , Witowski, J. , Adamos, A. , Major, P. , Nowakowski, M. , & Budzyński, A. (2018). Current status of enhanced recovery after surgery (ERAS) protocol in gastrointestinal surgery. Medical Oncology (Northwood, London, England), 35(6), 95. 10.1007/s12032-018-1153-0 29744679 PMC5943369

[nop21715-bib-0036] Polit, D. F. , & Beck, C. T. (2010). Generalization in quantitative and qualitative research: Myths and strategies. International Journal of Nursing Studies, 47(11), 1451–1458. 10.1016/j.ijnurstu.2010.06.004 20598692

[nop21715-bib-0037] Rossettini, G. , Conti, C. , Suardelli, M. , Geri, T. , Palese, A. , Turolla, A. , Lovato, A. , Gianola, S. , & Dell'Isola, A. (2021). COVID‐19 and health care leaders: How could emotional intelligence be a helpful resource during a pandemic? Physical Therapy, 101(9), pzab143. 10.1093/ptj/pzab143 34101807 PMC8418206

[nop21715-bib-0038] Sibbern, T. , Bull Sellevold, V. , Steindal, S. A. , Dale, C. , Watt‐Watson, J. , & Dihle, A. (2017). Patients' experiences of enhanced recovery after surgery: A systematic review of qualitative studies. Journal of Clinical Nursing, 26(9–10), 1172–1188. 10.1111/jocn.13456 27345939

[nop21715-bib-0039] Slamet, S. , Abdullah, I. , & Laila, N. Q. (2022). The contestation of the meaning of halal tourism. Heliyon, 8(3), e09098. 10.1016/j.heliyon.2022.e09098 35313489 PMC8933669

[nop21715-bib-0040] Smirk, A. J. , Nicholson, J. J. , Console, Y. L. , Hunt, N. J. , Herschtal, A. , Nguyen, M. N. H. H. , & Riedel, B. (2018). The enhanced recovery after surgery (ERAS) greenie board: A navy‐inspired quality improvement tool. Anaesthesia, 73(6), 692–702. 10.1111/anae.14157 29582421

[nop21715-bib-0041] Spinal cord Injury Prevention and Control Group, Spinal Disease Prevention and Control Committee, Chinese Preventive Medical Association& Basic Research Group, Spinal Cord Professional Committee, & Chinese Rehabilitation Association . (2019). Clinical guideline for perioperative management of acute spinal cord injury. Chinese Journal of Trauma, 7, 577–587. 10.3760/cma.j.issn.1001-8050.2019.07.001

[nop21715-bib-0042] Sun, H. H. , Chen, S. Q. , & Wang, X. H. (2019). Construction of quality evaluation index system of enhanced recovery nursing in general surgery department based on 3d quality structure model. Journal of Nursing Science, 26(23), 29–33. 10.16460/j.issn1008-9969.2019.23.029

[nop21715-bib-0043] Surgical Society of Chinese Medical Association & Chinese Society of Anesthesiology . (2018). Chinese consensus and clinical guidelines for enhanced recovery after surgery. Chinese Journal of Practical Surgery, 38(1), 1–20. 10.19538/j.cjps.issn1005-2208.2018.01.01

[nop21715-bib-0044] Thompson Burdine, J. , Thorne, S. , & Sandhu, G. (2021). Interpretive description: A flexible qualitative methodology for medical education research. Medical Education, 55(3), 336–343. 10.1111/medu.14380 32967042

[nop21715-bib-0045] Tossaint‐Schoenmakers, R. , Versluis, A. , Chavannes, N. , Talboom‐Kamp, E. , & Kasteleyn, M. (2021). The challenge of integrating eHealth into health care: Systematic literature review of the Donabedian model of structure, process, and outcome. Journal of Medical Internet Research, 23(5), e27180. 10.2196/27180 33970123 PMC8145079

[nop21715-bib-0046] Wang, D. , Liu, Z. , Zhou, J. , Yang, J. , Chen, X. , Chang, C. , Liu, C. , Li, K. , & Hu, J. (2022). Barriers to implementation of enhanced recovery after surgery (ERAS) by a multidisciplinary team in China: A multicentre qualitative study. BMJ Open, 12(3), e053687. 10.1136/bmjopen-2021-053687 PMC8921855 35288383

[nop21715-bib-0047] Whelan, A. , Halpine, M. , Christie, S. D. , & McVeigh, S. A. (2020). Systematic review of melatonin levels in individuals with complete cervical spinal cord injury. The Journal of Spinal Cord Medicine, 43(5), 565–578. 10.1080/10790268.2018.1505312 30132738 PMC7534275

